# Hypoxic Signaling in Skeletal Muscle Maintenance and Regeneration: A Systematic Review

**DOI:** 10.3389/fphys.2021.684899

**Published:** 2021-06-23

**Authors:** Tamara Pircher, Henning Wackerhage, Attila Aszodi, Christian Kammerlander, Wolfgang Böcker, Maximilian Michael Saller

**Affiliations:** ^1^Experimental Surgery and Regenerative Medicine, Department of General, Trauma and Reconstructive Surgery, Munich University Hospital, Munich, Germany; ^2^Faculty of Sport and Health Sciences, Technical University of Munich, Munich, Germany

**Keywords:** hypoxia-inducible factor 1 alpha, HIF1A, muscle regeneration, satellite cells, fusion

## Abstract

In skeletal muscle tissue, oxygen (O_2_) plays a pivotal role in both metabolism and the regulation of several intercellular pathways, which can modify proliferation, differentiation and survival of cells within the myogenic lineage. The concentration of oxygen in muscle tissue is reduced during embryogenesis and pathological conditions. Myogenic progenitor cells, namely satellite cells, are necessary for muscular regeneration in adults and are localized in a hypoxic microenvironment under the basal lamina, suggesting that the O_2_ level could affect their function. This review presents the effects of reduced oxygen levels (hypoxia) on satellite cell survival, myoblast regeneration and differentiation in vertebrates. Further investigations and understanding of the pathways involved in adult muscle regeneration during hypoxic conditions are maybe clinically relevant to seek for novel drug treatments for patients with severe muscle damage. We especially outlined the effect of hypoxia-inducible factor 1-alpha (HIF1A), the most studied transcriptional regulator of cellular and developmental response to hypoxia, whose investigation has recently been awarded with the Nobel price.

## Introduction

Since the great oxygenation event around 2.4 billion years ago, living organisms evolved in an oxygen-containing environment and many of today's species cannot survive without it. Oxygen (O_2_) plays a pivotal role in metabolic signaling, energy production and cellular homeostasis of aerobic organisms. At sea level, the atmospheric oxygen is ~21% in air generating a partial O_2_ pressure of about 160 mmHg. However, the physiological O_2_ levels in different tissues are significantly lower. For example, within skeletal muscle, the oxygen fraction generally varies between 2 and 10% (15–76 mmHg), depending on their location (Greenbaum et al., 1997).

A reduced level, tension or the deprivation of oxygen is termed hypoxia. For skeletal muscle tissues, the criteria for hypoxia are met if oxygen is below ≈2% (Yun et al., 2005). Hypoxia can be physiological as e.g., during embryogenesis (Dunwoodie, 2009), as a result of stimuli, such as high altitude and exercise (Sliwicka et al., 2017), or it can be caused by pathological defects, such as torn muscle fibers (Itoigawa et al., 2010) or peripheral arterial diseases (Norgren et al., 2007).

Skeletal muscles originate from somites along the rostro-caudal axis by fusion of myoblasts to mature myofibers. After embryogenesis, some myogenic progenitor cells (MPCs) remain quiescent and localize between the sarcolemma and the basal membrane. These cells are termed satellite cells and are the resident stem cells of skeletal muscle. Satellite cells are positive for paired-box transcriptional factor 7 (PAX7) but do not express myogenic differentiation 1 (MYOD1) (Zammit et al., 2004). Once activated by extrinsic factors, they express MYOD1 and become satellite cells-derived myoblasts, which regenerate muscle and contribute to muscle hypertrophy. Studies that stain satellite cell-related cells for markers such as PAX7 and MYOD1 have revealed the following cell types: PAX7^+^/MYOD1^−^ (quiescent and self-renewing satellite cells), PAX7^+^/MYOD1^+^ (activated, proliferating satellite cell-derived myoblasts) and PAX7^−^/MYOD1^+^/Myogenin^+^ (myoblasts that differentiate into multinuclear myotubes) (Liu et al., 2012).

During skeletal muscle development and regeneration, satellite cells are frequently exposed to hypoxia in their microenvironments. These so-called hypoxic niches have been reported to be critical for the regulation of satellite cell activation, self-renewal, proliferation and differentiation at the site of injured skeletal muscle (Parmar et al., 2007). This affects the activity of oxygen-sensing pathways, including the NOTCH-(Gustafsson et al., 2005), PI3K-AKT-mTOR- (Ren et al., 2010), MAPK14- (Ren et al., 2010) and HIF1α-depended pathways (Majmundar et al., 2015) and in turn these pathways then regulate hypoxia-induced responses. Amongst these pathways, the best investigated oxygen responsive factor is the transcription factor hypoxia-inducible factor 1α (HIF1A).

In this systematic review, we will discuss the molecular adaption of MPCs and satellite cells to hypoxia, focusing on RNA and protein level, as an understanding of the role of hypoxia in the myogenic lineage could be critical to develop hypoxia-related therapies for muscular conditions. To create the review, we searched for all data based on skeletal muscle tissue and hypoxia, excluding clinical trials (Supplementary Figure 1). The review was structured by the myogenic timeline of muscle cell regeneration, starting with self-renewal, followed by proliferation and finishing with differentiation.

## Methods

### Search Strategy

We searched *PubMed* and *Web of Science* for the keyword combinations shown in Table 1.

**Table 1:** Search Terms in PubMed and Web of Science for search strategy.

**Nr**.	**Search terms in *PubMed***		**Number of results**	**Date**
#1	[(muscle) AND Hypox*] AND regenerat*	All field	456	29.04.2019
#2	[(tissue engineering) AND Hypox*] AND regenerat*	All field	397	29.04.2019
#3	((myoblast*[Title/Abstract]) AND Hypox*[Title/Abstract]) AND Regen*[Title/Abstract]	Title/Abstract	30	29.04.2019
#4	((muscle[Title/Abstract]) AND Hypox*[Title/Abstract]) AND Regen*[Title/Abstract]	Title/Abstract	184	29.04.2019
**Nr**.	**Search terms in** ***Web of Science***		**Nr. of results**	**Date**
#1	Myoblast* AND hypox* AND regen*	All field	67	29.04.2019
#2	Muscle AND hypox* AND regen*	All field	551	29.04.2019
#3	Tissue engineering AND hypox* AND regen*	All field	797	29.04.2019
#4	Myoblast* AND hypox* AND regen*	Title	0	29.04.2019

Duplicates and articles in languages other than English were excluded from the obtained results. The remaining articles received with these keyword combinations were screened by their title and abstract, by excluding all clinical studies, studies including adipocytes, cardiac or smooth muscle cells. Residual manuscripts were screened by their full text, reviews, studies including immunological processes and further not skeletal muscle-related articles were excluded (Supplementary Figure 1).

### Data Analysis

All data included in the figures of the review were transferred to PathVisio (Version 3.3.0, Department of Bioinformatics, Maastricht University, Netherlands) for creating molecular interactions and pathways. All pathways are published online for free on WikiPathways.

## Results

### Hypoxia Keeps Satellite Cells in Their Undifferentiated State

Hypoxia regulates the identity and progression of satellite cells by regulating gene expression through NOTCH (Gustafsson et al., 2005) and WNT (Majmundar et al., 2015) signaling which we will discuss now.

**Figure 1:**
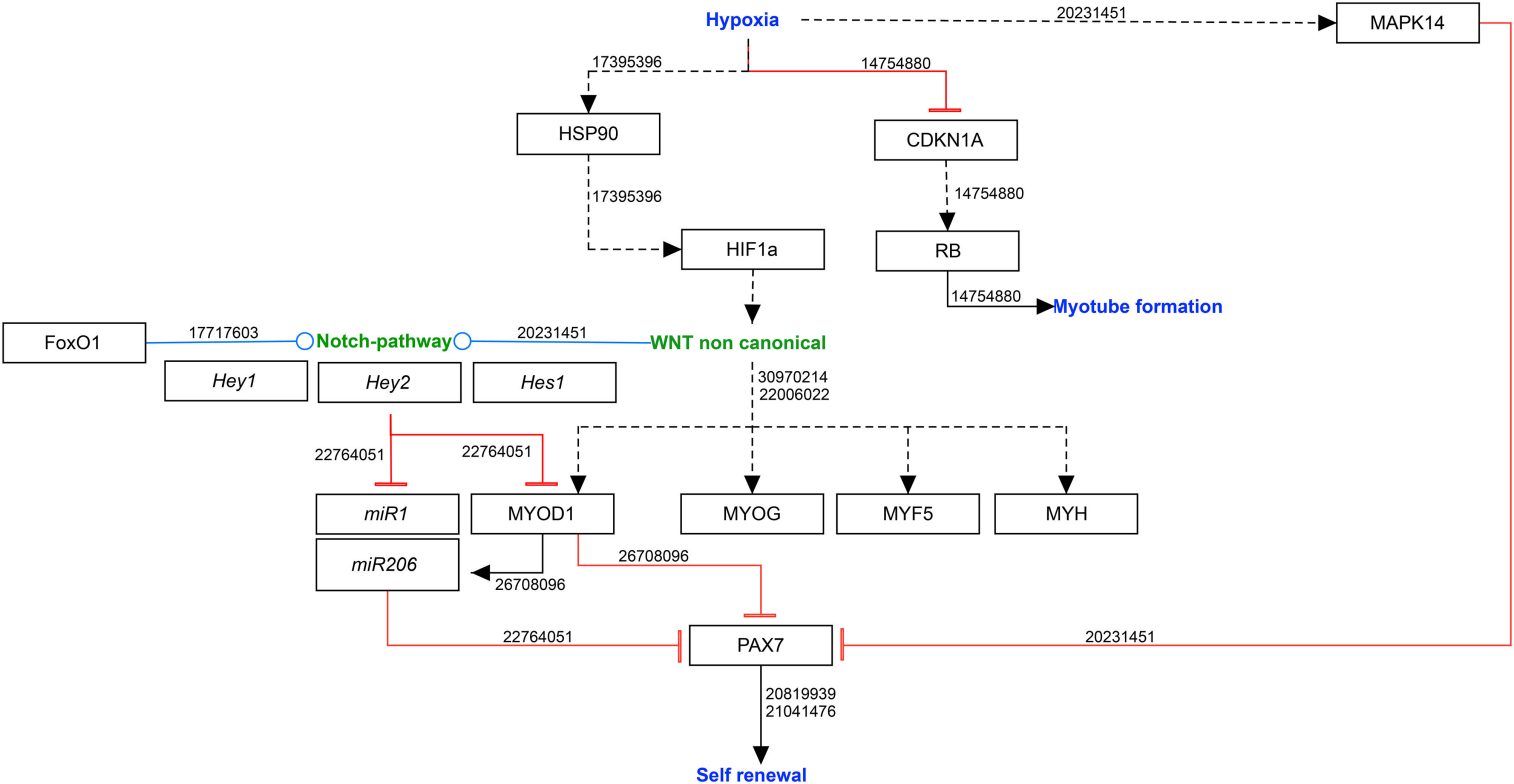
Molecular mechanisms involved in self-renewal of satellite cells in hypoxia. Black arrows: activation of the signaling pathway/protein/molecule. Blunt red arrow: inhibition of the signaling pathway/protein/molecule. Round blue arrow: interaction between two pathways. Numbers: PubMed-IDs. https://www.wikipathways.org/index.php/Pathway:WP5023.

Under normoxia, microRNA1 and 206 (*miR1* & *miR206*) downregulate PAX7 protein production leading to reduced self-renewal of the satellite cells (Chen et al., 2010; Dey et al., 2011), hypoxia allows PAX7 to regulate asymmetric self-renewal division of satellite cells, by reducing *miR1* & *miR206* expression. Thereby, hypoxia ensure a sufficiently large stem cell pool without affecting the overall proliferation (Liu et al., 2012). This mainly occurs due to activation of NOTCH and non-canonical WNT signaling pathways (Figure 1).

#### NOTCH Signaling Pathway

The NOTCH signaling pathway is not only activated during embryonic muscle development to promote cell division, but also contributes to regeneration during muscle injury, which is associated with hypoxia as blood vessels are damaged. Gustafsson et al. reported a downregulation of myogenic differentiation in hypoxia through the activation of NOTCH signaling pathway, leading to an upregulation of HEY2 (hes related family bHLH transcription factor with YRPW motif 2) and therefore maintaining the myogenic cells in their undifferentiated state (Gustafsson et al., 2005). Acute hypoxia promotes the interaction between FOXO1 (forkhead box O1 transcription factor) and the NOTCH pathway upregulating the expression of NOTCH downstream transcription factor genes *Hes1* (hes family bHLH transcription factor 1) and *Hey1* (Kitamura et al., 2007). Furthermore, hypoxia promotes the division of primary murine myoblasts by a NOTCH-dependent upregulation of *Hes1/Hey1*, which in turn lead to a suppression of *miR1* and *miR206* in a *MyoD1*-independent manner (Liu et al., 2012).

#### Non-canonical WNT Signaling Pathway

The canonical and non-canonical WNT signaling pathways are major regulators of myogenesis (Chen et al., 2005; Borello et al., 2006). Of these, mainly the non-canonical WNT mediates the cellular response to hypoxia (Cirillo et al., 2017). While canonical WNT signaling mediates satellite cell division, non-canonical WNT signaling regulates the symmetric expansion of satellite cells and myofiber growth, resulting in hypertrophy (von Maltzahn et al., 2011). Low oxygen levels trigger non-canonical WNT signaling through at least two mechanisms: via protein kinase C (PKC), which activates calcium/calmodulin-dependent protein kinase II (CAMKII) and the RHOA/JNK (Ras homolog family member A/**c-**Jun N-terminal kinases) pathways, which leads to ATF/CREB (activating transcription factors/cAMP response element binding protein) activation (Veeman et al., 2003). Previous studies revealed *Wnt7, Wnt9a* and *Wnt4* as important players in non-canonical pathway (Cirillo et al., 2017) and WNT4 upregulation leads to an increase of myogenin expressing cells (Leroux et al., 2010).

#### CDKN1A/B (p21/p27)

Although the main effect of non-canonical WNT signaling is the inhibition of differentiation, differentiating cells need to withdraw from the cell cycle, which is regulated by cyclin-dependent kinases (CDKs) and their inhibitors (CDKIs).

Cyclin-dependent kinase inhibitor 1 (CDKN1A, also known as p21) is a major target of TP53 and regulates cell cycle progression by inhibiting cyclin/CDK complexes (Di Carlo et al., 2004). Additionally, it leads to the accumulation of the tumor suppressor retinoblastoma protein (RB), which is necessary to establish a permanent post-mitotic state in myogenesis (Schneider et al., 1994; Novitch et al., 1996). In severe hypoxia (<1% oxygen), however, the CDKN1A as well as the RB accumulation is inhibited (Di Carlo et al., 2004).

### Effects of Hypoxia on Proliferation of Skeletal Muscle Cells

Hypoxia with 2% oxygen stimulates the proliferation of mouse satellite cells (Urbani et al., 2012) and human primary myoblasts (Koning et al., 2011) *in vitro* by increasing the expression of the myogenic transcription factors MYF5 and MYOD1 (Koning et al., 2011).

Through induction of muscle-specific gene expression, quiescent satellite cells start to proliferate and subsequently differentiate into myotubes. The main muscle regulatory transcription factors (MRFs) are MYOD1, myogenic factor 5 (MYF5), myogenin (MYOG) and myogenic factor 6 (MYF6). Upon activation and proliferation, MYF5 and MYOD1 expression is upregulated in satellite cells and their expression remains high until late into differentiation (Megeney et al., 1996; Cornelison and Wold, 1997; Cooper et al., 1999).

Ogilvie et al. (2000) reported an effect of the hypoxia-induced hormone erythropoietin (EPO) on the proliferation of C2C12 cells and primary satellite cells. They observed that EPO could stimulate the proliferation of myoblasts and inhibit their differentiation by inducing *Myf5* and *MyoD1* expression (Ogilvie et al., 2000). Similar results were reported by Jazwa et al. who observed an influence of the hypoxia-regulated HMOX1 (heme oxygenase 1) on MRFs by increasing expression of *MyoD1* and *MyoG* under ischemic conditions (Kozakowska et al., 2012; Jazwa et al., 2013). Jia et al. reported that EPO stimulates the recruitment or proliferation of satellite cells in injured muscle. Their data indicate that endogenous EPO may promote satellite cell survival in skeletal muscles by an increased expression of phosphatidylinositol 3 kinase (PI3K)-AKT-associated proteins (Jia et al., 2012).

**Figure 2:**
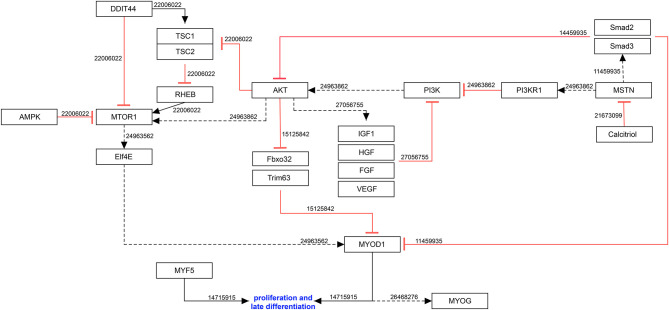
Molecular mechanisms involved in myogenic proliferation during hypoxia. Black arrow: activation of the signaling pathway/protein/molecule. Blunt red arrow: inhibition of the signaling pathway/protein/molecule. Numbers: PubMed-IDs. https://www.wikipathways.org/index.php/Pathway:WP5024.

In addition, GATA transcription factors can regulate differentiation, growth, and survival of a wide range of cell types (Molkentin, 2000). In myoblasts, *GATA3* overexpression increases proliferation of undifferentiated C2C12 cells and can contribute to the proliferative response of EPO (Jia et al., 2012). In addition, it has been shown that the upregulated EPO in hypoxic conditions induces *GATA3* and enables the activation of JAK2/STAT5a (Janus kinase 2/signal transducer and activator of transcription 5A) pathways (Jia et al., 2012), which is upregulated during early myogenic differentiation *in vitro* (Wang et al., 2008) (Figure 2).

#### Negative Feedback-Loop of Proliferation Controls Muscle Growth and Differentiation

Hypoxia also interferes with the negative feedback-loop of proliferation. For example, hypoxia causes myoblasts to release the transforming growth factor-β superfamily member myostatin (*MSTN*) (Hayot et al., 2011), which inhibits proliferation and enhances muscle growth by reducing protein degradation (Hauerslev et al., 2014).

Upregulation of MSTN leads to a negative loop which decreases the expression of MYOD1 (Langley et al., 2002) by binding to the ActRIIb (activin receptor type-2B) and activating SMAD2 (SMAD family member 2), which directly inhibits MYOD1 through AKT signaling inhibition (Lee and McPherron, 2001). During proliferation, AKT regulates the translation of MYOD1 (Hauerslev et al., 2014) by preventing the degradation of MYOD1 in FBXO32 (F-box protein 32) and TRIM63 (tripartite motif containing 63-dependent manner (Stitt et al., 2004). Altogether, MSTN reflects an oxygen-dependent key molecule in the regulation of myogenesis, especially during proliferation and differentiation (Rios et al., 2002).

#### HDAC9 Suppresses Myoblast Differentiation in Hypoxia

A recent study reported an increased expression of histone deacetylases (HDACs) including *Hdac1, Hdac5, Hdac8* and *Hdac9* in C2C12 cells after exposure to hypoxia (Zhang et al., 2019). The study mainly focused on HDAC9 and reported enhanced myogenesis and upregulation of MyoG and MyoD1 by *Hdac9* inhibition. Additionally, the HDAC proteins were linked to autophagy by demonstrating a hypoxia-dependent enrichment of HDAC9 binding in the promoter region of autophagy-related genes *Atg7, Becn1, Map1lc3a, and Map1lc3b* (Zhang et al., 2019). Autophagy results in phosphorylation of GSK3ß (glycogen synthase kinase 3 beta) and was shown to directly regulate the canonical WNT pathway. Under hypoxia, phosphorylated GSK3ß and activated ß-catenin are both decreased and thus, myogenesis is impaired. These results imply that canonical Wnt signaling is negatively regulated by HDAC9 activation (Zhang et al., 2019).

#### PI3K-AKT Signaling Pathway Repression at Low Oxygen Levels Impedes Myoblast Differentiation

The PI3K-AKT-mTOR pathway, which participates in myogenic differentiation is downregulated due to oxygen deprivation (Ren et al., 2010). As described earlier, the PI3K-AKT signaling pathway prevents MYOD1 degradation and stimulates MYOD1 translation due to mTOR1 activation. Once the oxygen level drops, it stimulates the eukaryotic translation initiation factor 4E (ELF4E), which is normally bond to its repressor: the 4E-binding protein (Hauerslev et al., 2014). MTOR (mammalian target of rapamycin) depends on RHEB (Ras homolog enriched in brain) activation (Li et al., 2007). This Ras-related small GTPase is regulated within the cell by tumor-suppressant proteins that form the tuberous sclerosis complex (TSC) (Li et al., 2007). Under physiological condition, those complexes are inhibited by AKT, but when cells are exposed to stress or hypoxia, AMPK (AMP-activated protein kinase) and DDIT4 (DNA-damage-inducible transcript 4) pathways are activated, which inhibit RHEB (Wouters and Koritzinsky, 2008).

The PI3K-AKT signaling pathway is partially down regulated due to a reduced IGF-IR (IGF-I receptor) sensitivity at low oxygen levels (Majmundar et al., 2012).

#### Hypoxia, ROS- and Oxygen-Dependent microRNAs Regulation

The expression of small non-coding microRNAs (miRs), post-transcriptional regulators, can be ROS- as well as hypoxia - and oxygen-dependent. Accordingly, *miR210* can also modulate the generation of reactive oxygen species (ROS) (Cicchillitti et al., 2012).

In myogenesis, eight miRs are currently known to be muscle tissue-specific and involved in myogenic processes: *miR1, miR206, miR133A/B, miR208A/B, miR486, miR499* (Horak et al., 2016), *miR26A* (Lee et al., 2015) and *miR210* (Cicchillitti et al., 2012), which are all controlled by MRFs. For example, *miR1* and *miR206*, as described earlier, target *PAX3* and *PAX7* mRNA, respectively, and are both activated by MYOD1 (Horak et al., 2016). Together with *miR133, mirR1* is also suggested to be required for proper somitogenesis (Chen et al., 2006). Additionally, there was the realization that some miRNAs are expressed tissue specific. Heart and skeletal muscle specific miRNA are called myomiR, including miR1, miR133 (Sempere et al., 2004), miR208A/B, miR499 and miR486 (van Rooij et al., 2007, 2009; Small et al., 2010). MiR206 holds a special role, since it is exclusively expressed in skeletal muscle cells (McCarthy et al., 2007). Moreover, changes in miR208b and miR499 expression during muscle atrophy showed a role in skeletal muscle plasticity (McCarthy et al., 2009).

One of the major targets activated by VEGF (vascular endothelial growth factor) during hypoxia is *miR210*. Even though *miR210* has no influence on myotube number or fusion, it provides myotube survival in presence of mitochondrial dysfunction and oxidative stress by modifying mitochondrial metabolism through the modulation of ROS regeneration and direct repression of apoptosis-related genes such as CASP8AP2 (CASP8-associated protein 2) (Kim et al., 2009).

### Effects of Hypoxia on Myogenic Differentiation

Hypoxia inhibits the differentiation of myoblasts. In contrast to self-renewal and proliferation, myogenic differentiation is divided into early and late phases, which are partly represented by different active molecules. During early differentiation, MYF5 and MYOD1 function as active regulatory muscle transcription factors. MYOD1 removes cells from the cell cycle and enhances the transcription of MYOG, which dominates the late differentiation, together with MRF4 (Zhou and Bornemann, 2001).

In 2004, Di Carlo et al. investigated the effect of severe hypoxia (<1% O_2_) on myogenic differentiation of C2C12 myoblasts. They observed growth arrest, and inhibition of MRFs expression and myogenic differentiation, due to decreased RB, CDKN1A, MYOD1, MYOG and MYHC1 (myosin heavy chain 1) and increased CDKN1B (Di Carlo et al., 2004). While hypoxic cultured myoblasts showed significantly impaired proliferation and differentiation (Di Carlo et al., 2004), Cirillo et al. described an upregulation of various MRFs due to hypoxic preconditioning of myogenic cells. Preconditioning induced a decrease of MYOD1 inhibition through downregulation of ID1 (inhibitor of differentiation/DNA binding 1) and MYOR (myogenic repressor) (Cirillo et al., 2017). The mitogen-activated protein kinase family member 14 (MAPK14) is involved in the terminal differentiation of myoblasts by activating transcription factors, such as MYOD1 and the myocyte enhancer factor-2 (MEF2). Hypoxia induces a decrease of p38-mediated phosphorylation in differentiating myoblasts, thus contributing to the initialization of the switch from differentiation to proliferation (Ren et al., 2010).

#### Epigenetic Regulation of Myogenic Cell Differentiation in Hypoxia

Recently, it was discovered that the histone demethylase KDM6A (lysine demethylase 6A) prevents cell differentiation by demethylation of H3K27 (histone H3 on lysine 27). Hypoxia can induce H3K27 methylation, which represses the transcription of late myogenic genes such as *MyoG*. The differential oxygen affinities of KDM6A and KDM6B suggests that the ability to promote H3K27 methylation and block differentiation is caused specifically by a loss of KDM6A activity within hypoxia. Indeed, the down-regulation of KDM6A, but not KDM6B, with different short hairpin RNAs phenocopied the effects of hypoxia on differentiation. Therefore, Abhishek et al. tried to directly increase KDM6A's oxygen affinity. By comparing the catalytic JmjC domains of KDM6A and KDM6B, they noted two non-conserved residues M^1190^ (KDM6A) → T^1434^ (KDM6B) and E^1335^ (KDM6A) → D^1579^ (KDM6B). A variant of KDM6A harboring these two KDM6B-like changes showed a 2-fold increased affinity of oxygen *in vivo* and was superior to the native KDM6A at rescuing differentiation under hypoxic conditions (Chakraborty et al., 2019).

#### Hypoxia-Induced Factor 1 and 2 Alpha

Hypoxia-inducible factor 1 alpha (HIF1A) is the most studied transcriptional regulator of the cellular and developmental response to hypoxia. For this reason, the Nobel Prize in Physiology and Medicine 2019 was awarded to the tree physician scientists who discovered and characterized HIF1, i.e., William G. Kaelin, Jr., Peter Ratcliffe and Gregg Semenza. Collectively, they have demonstrated that the response of gene expression to oxygen availability is directly coupled to oxygen levels in the animal cell, allowing immediate cellular responses to occur for oxygenation through the action of the HIF1 transcription factor complex.

The heterodimeric HIF1 transcription factor is under control of the oxygen-activated prolyl hydroxylase domain containing enzymes (PHD1, PHD2, PHD3), which act as oxygen sensors. The most common described prolyl hydroxylase is PHD2 (gene symbol *EGLN1*). In the presence of oxygen, the PHD-mediated prolyl hydroxylation leads to an ubiquitination and subsequent proteasomal degradation of HIF1A (Marxsen et al., 2004). During oxygen deprivation, the alpha-subunit is stabilized by the heat shock protein (HSP) HSP90 to prevent degradation (Kubis et al., 2005; Ono et al., 2006). Once stabilized, the alpha-subunit translocates to the nucleus and forms a heterodimer with its beta subunit. The newly formed complex bind to hypoxia response elements (HREs) in the promoter region of target genes, such as: genes to increase oxygen capacity in the blood (*EPO, HMOX1*), proangiogenic genes (*VEGF, ADM*) and metabolic genes associated with glycolysis and glucose uptake (Bracken et al., 2003).

Moreover, HIF1A activation, as well as hypoxia itself, induce BHLHE40 (Class E basic helix-loop-helix protein 40), a protein that is part of the TP53 signaling pathway. It was shown to repress myogenic differentiation by binding to the E-box sequence of the *MyoG* promoter, leading to reduced transcriptional activity of MyoD1 on *MyoG* (Wang et al., 2015). Moreover, HIF1A directly inhibits the canonical WNT signaling pathway and various MRFs, such as MYF5, MYOD1, MYOG, MRF6 and MYH (myosin heavy chain), leading to a repression of myogenic proliferation and differentiation (Sinha et al., 2019).

Another important member of the hypoxia-inducible transcription factors is HIF2A. Contrary to HIF1A, which is mainly expressed in myoblasts and regulates proliferation in hypoxic conditions, HIF2A is mainly expressed in quiescent SCs. Xie et al. did not detect HIF1A in PAX7^+^ quiescent satellite cells, whereas over 90% of these cells were HIF2A positive (Xie et al., 2018). Its major roles include enhancing the slow myofiber formation through acting downstream of peroxisome proliferator-activated receptor-γ coactivator 1α (PGC-1α) (Rasbach et al., 2010) and promoting stemness of satellite cell (Liu et al., 2012; Majmundar et al., 2012). Leukemia inhibitor factor (LIF) stimulates myoblast growth and fusion *in vivo, a*cting directly on the side of injury and stimulating hypertrophy (Barnard et al., 1994; White et al., 2001). In addition, it was shown that HIF2A can directly activate the expression of LIF in colorectal cancer (Wu et al., 2015). However, it is not clear, if the mechanism through HIF2A activation also occurs in skeletal muscle regeneration.

Through a *Pax7*-specific double knockout of *Hif1a* and *Hif2a* in mice, Yang et al. could show the necessity of both transcription factors in self-renewal of satellite cells within hypoxic environments. While in normoxia a knockout of *Hif1a*/*Hif2a* did not affect muscle stem cell function, an oxygen level <1% showed decreased self-renewal and increased differentiation of myoblasts, without effects on the proliferation (Yang et al., 2017).

In skeletal muscle, both hypoxia-inducible transcription factors have a pro-angiogenetic function by inducing VEGF expression and the development of capillaries that express CD31 *in vivo* (Niemi et al., 2014). VEGF is not only essential for angiogenesis but also for skeletal muscle regeneration (Germani et al., 2003), even though it could have pro-fibrotic effects by inducing stress fiber formation in muscle-specific fibroblasts of dystrophic muscles (Gutpell and Hoffman, 2015).

**Figure 3:**
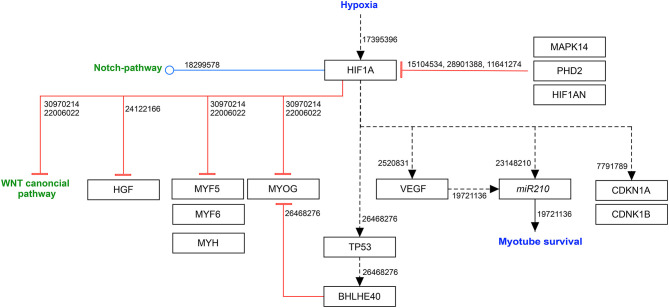
HIF1A modulates myogenic differentiation in hypoxia. Black arrow: activation of the signaling pathway/protein/molecule. Blunt red arrow: inhibition of the signaling pathway/protein/molecule. Round blue arrow: Interaction between two pathways. Numbers: PubMed-IDs. https://www.wikipathways.org/index.php/Pathway:WP5025.

In addition, HGF (hepatocyte growth factor) has been shown to have a major impact on myogenesis, as well as on adult regeneration. It leads to an upregulation of several signals such as the AKT signaling pathway (Rosova et al., 2008) and the CDKN1A (Hauerslev et al., 2014). Intriguingly, the promoter region of *Hgf* has a HRE element, and thus, binding of HIF1A leads to a downregulation of HGF in hypoxia (Flann et al., 2014) (Figure 3).

The p38 signaling pathway has been shown to inhibit HIF1A expression. Thereby, HIMGB1 (high mobility group protein B1) is be involved in the activation of MAPK14 (p38) through RAGE (receptor for advanced glycation end-products) (Sorci et al., 2004).

Li et al. revealed an impact of estradiol (E2) on MAPK14. In a hypoxic environment, estradiol is active and binds to the estrogen receptor alpha (ERα), leading to MAPK14 phosphorylation (Li et al., 2017). MAPK14 additionally affects the activation of MYOD1 and MEF2 (myocyte enhancer factor-2) during differentiation (Keren et al., 2006).

HIF1AN (hypoxia-inducible factor 1-alpha inhibitor), an alpha-ketoglutarate-dependent dioxygenase, is the key element of negative loop regulation of HIF1A by hydroxylating the C-terminal transactivation domain of HIF1A. In hypoxia, HIF1AN is downregulated to allow HIF1A stabilization (Mahon et al., 2001). It also works as a negative regulator of NOTCH, which underlines the link between both pathways (Zheng et al., 2008).

In C2C12 cells *in vitro*, the inhibition of HIF1A leads to a decreased myoblast fusion, which is accompanied by a reduction of *MyoG* and *Myh* expression (Cirillo et al., 2017).

## Summary and Conclusion

A reduction of oxygen levels occurs during embryogenesis and in several muscular diseases and is necessary for normal development and muscle regeneration. Cell culture studies demonstrate that myogenic differentiation is improved at oxygen levels of 2–10%. This mimics the physiological oxygen concentration in skeletal muscle tissue. Furthermore, a hypoxic preconditioning in <1% O_2_ has a positive effect on muscle regeneration. However, if myoblasts remain in an environment of <1% O_2_ over a longer period of time, it has negative and even toxic effects on myoblasts leading to apoptosis.

Generally, hypoxia prevents cell differentiation, as well as myotube growth, in muscular tissue by mainly influencing MRFs and is essential to keep satellite cells in their quiescent state. The most investigated factor that acts in low oxygen concentrations is HIF1A, which mediates the inhibition of myogenesis by involving WNT and NOTCH signaling pathways. In hypoxic environment, myogenic differentiation is additionally repressed by the inhibition of AKT/mTOR pathway as well as the induction of BHLHE40. Numerous factors and signaling pathways have proposed to modulate myogenesis during hypoxia, however this review only includes a small section, focusing onto protein and mRNA level. Metabolic and functional impacts were not discussed. Although, a deeper understanding and holistic comprehension is necessary to treat patients with extensive muscle damage associated with hypoxia.

## Data Availability Statement

Publicly available datasets were analyzed in this study. This data can be found here: https://www.wikipathways.org/index.php/Pathway:WP5023; https://www.wikipathways.org/index.php/Pathway:WP5024; https://www.wikipathways.org/index.php/Pathway:WP5025.

## Author Contributions

TP was part of the acquisition of the literature, was substantial contributor in developing the conception and analyzed the data. HW was substantial contributor in developing the conception and drafting of the manuscript. AA, CK, and WB substantively revised the manuscript. MS was part of the acquisition of the literature, substantially contributed to the developing the conception and drafting of the manuscript. All authors contributed to the article and approved the submitted version.

## Conflict of Interest

The authors declare that the research was conducted in the absence of any commercial or financial relationships that could be construed as a potential conflict of interest.

## Supplementary Material

Supplementary Figure 1 PRISMA flow diagram for the systematic review.
