# Supplementary figures and images for: Hypoxic Signaling in Skeletal Muscle Maintenance and Regeneration: A Systematic Review
Source: Front Physiol. 2021 Jun 23;12:684899. doi: 10.3389/fphys.2021.684899 (PMC8260947; doi:10.3389/fphys.2021.684899)

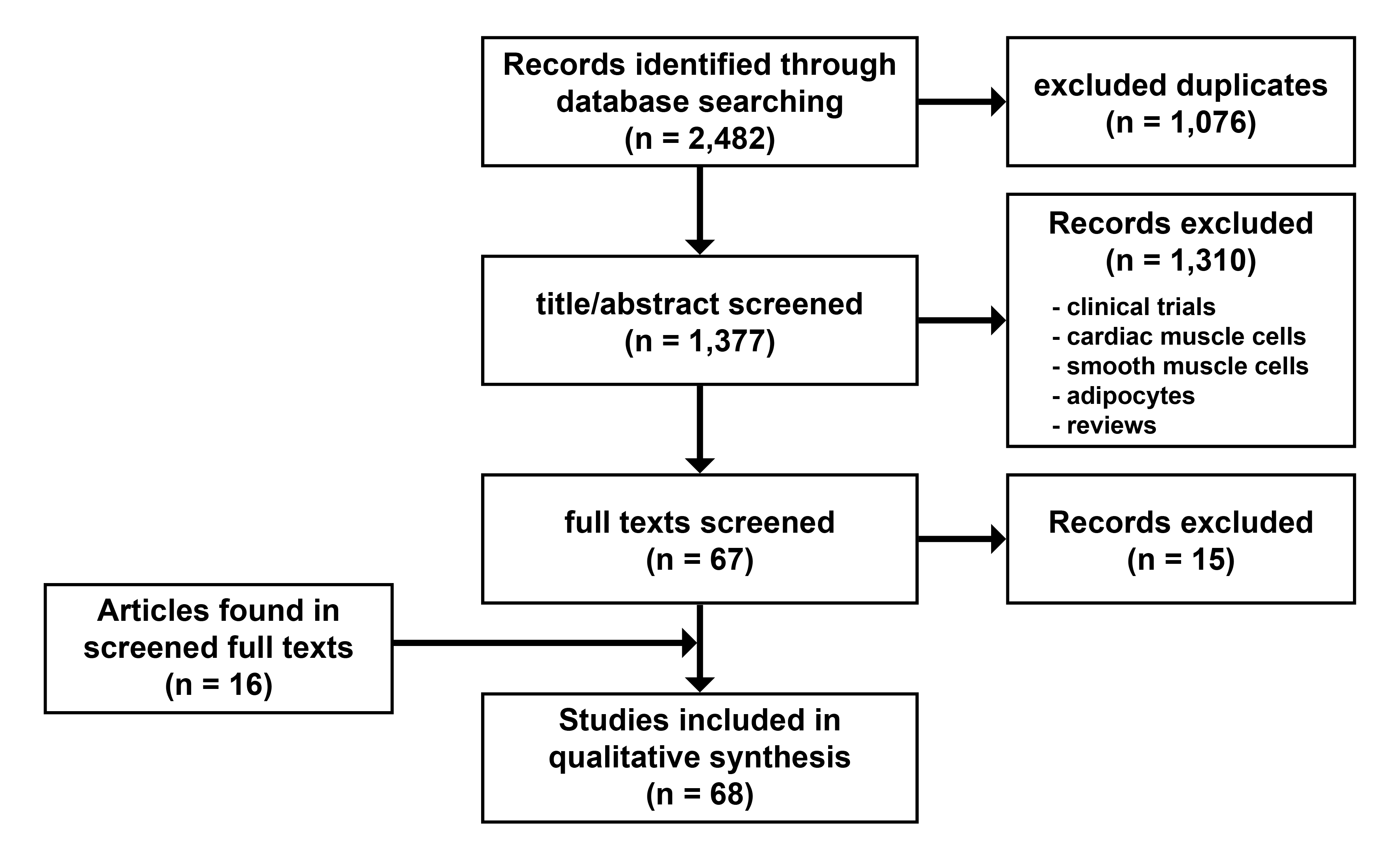

Supplement: Supplementary Figure 1 — PRISMA flow diagram for the systematic review. [file Image_1.TIF]
